# Stochastic allelic expression as trigger for contractile imbalance in hypertrophic cardiomyopathy

**DOI:** 10.1007/s12551-020-00719-z

**Published:** 2020-07-13

**Authors:** Judith Montag, Theresia Kraft

**Affiliations:** Molecular and Cell Physiology, Hannover Medical School, Hannover, Germany

**Keywords:** Hypertrophic cardiomyopathy, Contractile imbalance, Burst-like transcription, Cell-to-cell allelic imbalance

## Abstract

Hypertrophic cardiomyopathy (HCM), the most common inherited cardiac disease, is caused by several mostly heterozygous mutations in sarcomeric genes. Hallmarks of HCM are cardiomyocyte and myofibrillar disarray and hypertrophy and fibrosis of the septum and the left ventricle. To date, a pathomechanism common to all mutations remains elusive. We have proposed that contractile imbalance, an unequal force generation of neighboring cardiomyocytes, may contribute to development of HCM hallmarks. At the same calcium concentration, we found substantial differences in force generation between individual cardiomyocytes from HCM patients with mutations in β-MyHC (β-myosin heavy chain). Variability among cardiomyocytes was significantly larger in HCM patients as compared with donor controls. We assume that this heterogeneity in force generation among cardiomyocytes may lead to myocardial disarray and trigger hypertrophy and fibrosis. We provided evidence that burst-like transcription of the *MYH7*-gene, encoding for β-MyHC, is associated with unequal fractions of mutant per wild-type mRNA from cell to cell (cell-to-cell allelic imbalance). This will presumably lead to unequal fractions of mutant per wild-type protein from cell to cell which may underlie contractile imbalance. In this review, we discuss molecular mechanisms of burst-like transcription with regard to contractile imbalance and disease development in HCM.

## Introduction

Hypertrophic cardiomyopathy (HCM) is characterized by asymmetric hypertrophy of the interventricular septum and the left ventricular wall. At the cellular level, often pronounced disarray of cardiomyocytes and myofibrils and increased fibrosis is found in the myocardium (Maron and Maron 2013). Clinically, the disease is highly heterogeneous, ranging from mild and almost asymptomatic disease courses to severe diastolic dysfunction or heart failure. HCM is the most common cause of sudden cardiac death in young athletes (Maron and Maron 2013). The prevalence of HCM is generally assumed to be 1:500 (Maron et al. 2012); recent publications even estimate a prevalence of 1:200 (Semsarian et al. 2015).

Several different mutations in an increasing number of genes have been associated with HCM. Almost all patients are heterozygous for the respective mutation; the disease is transmitted autosomal dominant (Maron et al. 2012). According to the European Society for Cardiology, 40–60% of the HCM patients encode for mutations that affect sarcomeric proteins (Authors/Task Force et al. 2014). Among these, *MYH7*, encoding for the β-myosin heavy chain (β-MyHC) and *MYBPC3*, encoding for the cardiac myosin binding protein C (cMyBP-C) account for more than 80%. Another 10% is covered by *TNNT2* and *TNNI3*, encoding for cardiac troponin T (cTnT) and troponin I (cTnI), respectively (Walsh et al. 2017). HCM is mostly a monogenic disorder; patients usually encode for one causative mutation. However, rare cases of patients encoding for two distinct mutations in the same gene on different alleles (compound heterozygous) or two mutations in different genes (double heterozygous) have been reported. These patients often exhibit a much more severe course of disease (Richard et al. 2003; Van Driest et al. 2004). In addition, the emergence of high throughput sequencing methods enables the comprehensive analysis of single nucleotide variants (SNV). This led to the detection of SNVs that are frequent in the healthy population but may trigger susceptibility to HCM if patients cumulate two or more of these SNVs (Burns et al. 2017; Thomson et al. 2019).

To date, no common mechanism has been determined to explain how different mutations in different genes can lead to the same phenotype of HCM. Patients with missense mutations, the most common type of HCM mutations (Maron et al. 2012), express both mutant and wild-type mRNA and protein. According to the *poison peptide hypothesis*, HCM mutations alter the physiological function of the respective proteins, thereby affecting the force generating mechanism in cardiomyocytes (Bonne et al. 1998). Depending on the affected gene and on the localization within the gene, mutations can alter calcium sensitivity, isometric force levels, acto-myosin ATPase activity, shortening velocity, relaxation properties, and/or cross-bridge cycle kinetics (reviewed in (Ashrafian et al. 2011; Marian and Braunwald 2017; Moore et al. 2012)). Interestingly, alterations can be highly divergent. *MYH7*-mutations R719W and R723G e.g. decrease calcium sensitivity (Kirschner et al. 2005) whereas *MYH7*-mutation I736T (Kirschner et al. 2005) or TnT-mutation R92Q (Robinson et al. 2002) increase calcium sensitivity. ATPase rates can either be increased (*MYH7*-mutation R719W (Seebohm et al. 2009)) or reduced (*MYH7*-mutation R453C (Bloemink et al. 2014; Sommese et al. 2013)). This exemplary listing may depict the complexity of HCM mutation effects on sarcomeric function. In addition, the poison peptide mechanism likely does not apply to mutations that lead to a truncated protein, as many mutations in *MYBPC3* do. In most patients, the truncated isoform is not incorporated into sarcomeres, the total level of functional cMyBP-C is reduced in cardiomyocytes and originates from the wild-type allele only (*haploinsufficiency*) (Vignier et al. 2009). The cMyBP-C is a structural protein that interacts with the thick and the thin filament and seems to act as an inhibitor of cross-bridge interactions (van Dijk et al. 2012). Reduced levels of cMyBP-C in HCM patients with haploinsufficiency presumably lead to alterations in force generation, especially altered cross-bridge cycle kinetics (reviewed in (Marian and Braunwald 2017; Schlossarek et al. 2011)). Taken together, a common mechanism of HCM development caused by different mutations remains elusive.

Evidence from our own previous work, however, led us to suggest a concept for a potential common mechanism that contributes to HCM development for heterozygous mutations which alter parameters of cardiomyocyte force generation. When we analyzed the effects of *MYH7* mutations in single *M. soleus* fibers, we detected a large variability in calcium-dependent force generation among individual fibers from the HCM patients, respectively (Kirschner et al. 2005). The variability was substantially larger than between fibers from control individuals. We also observed a similarly large variability among individual cardiomyocytes from HCM patients with different mutations in the *MYH7* gene, respectively (Montag et al. 2018). We hypothesized that different fractions of mutated and wild-type protein from cell to cell might be the reason for the observed highly variable function of individual muscle cells (Brenner et al. 2014; Kirschner et al. 2005; Kraft et al. 2016; Montag et al. 2018). A mosaic of stronger and weaker cells may thus lead to contractile imbalance between individual cardiomyocytes (Brenner et al. 2014; Kraft et al. 2016; Montag et al. 2018). This hypothesis was supported by the finding that fractions of mutant and wild-type mRNA varied substantially among individual HCM cardiomyocytes from the same cardiac tissue which had been used in functional studies (Kraft et al. 2016; Montag et al. 2018). As underlying mechanism that may lead to the observed unequal allelic expression of *MYH7*, we identified stochastic, burst-like expression which is independent for mutant and wild-type alleles (Montag et al. 2018). The resulting c*ontractile imbalance* may disrupt the functional syncytium of the myocardium and contribute to development of myocardial disarray, hypertrophy, and fibrosis. In this review, we aim to further elucidate the mechanisms that underlie contractile imbalance and how it may affect disease development in HCM.

## Stochastic gene expression from cell to cell

Several years ago, researchers detected in bacteria and yeast that cells, which originate from the same genetic background, express divergent levels of specific proteins. In a culture of clonal cells, some cells expressed low levels, some expressed high levels, and some expressed medium levels of the identical protein. This evoked a phenotypic variability between individual cells (Blake et al. 2003; Elowitz et al. 2002) that resulted in a mixture of cells with different functional properties. Interestingly, over time, protein expression in individual cells changed and likewise did the functional activity (Cai et al. 2006). Such a phenotypic heterogeneity was subsequently also shown in cultured mammalian cells (Lo et al. 2015; Sigal et al. 2006) and cancer cell lines (Roumeliotis et al. 2017).

**Fig. 1:**
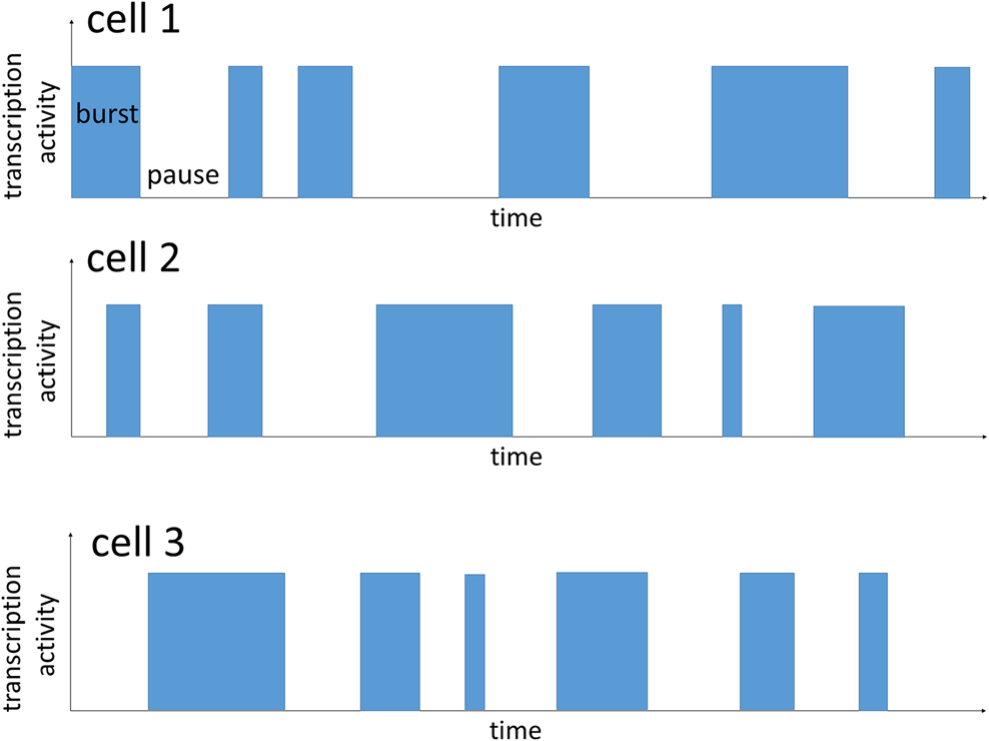
The transcriptional activity of three individual cells is schematically depicted. A burst of transcription is indicated by a blue bar, transcriptional pauses are indicated by the absence of bars. At a given time point, some cells are in a bursting phase whereas in other cells transcription is pausing. This will lead to different mRNA and protein amounts in the cells, which may cause phenotypic variability between the individual cells

The variable protein expression from cell to cell and over time was attributed to the so-called *burst-like transcription*. This model of gene expression assumes that transcription occurs in stochastic pulses (Blake et al. 2003; Elowitz et al. 2002). Transcription of a gene starts, pauses, and restarts again in a stochastic manner over time (Fig. 1). Burst-like transcription has been determined in bacteria, yeast, and in mammalian cells, including cells within tissues (Blake et al. 2003; Elowitz et al. 2002; Montag et al. 2018; Raj et al. 2006; Raj and van Oudenaarden 2008). In a tissue, this means that one cell actively transcribes a respective gene while in a neighboring cell transcription can pause at the same time (Fig. 1). Even though accumulation of mRNA from recent bursts within the cells will partly equalize total mRNA and protein levels from cell to cell, a distinct variability between cells has been reported to remain (Blake et al. 2003; Cai et al. 2006; Elowitz et al. 2002; Symmons et al. 2019). To minimize functional differences between cells, the stochasticity of on and off switches appears low for structural proteins to gain comparable levels of proteins from cell to cell and allow for concerted actions of the tissue (Rajapakse and Smale 2017; Sun and Zhang 2020). In contrast, it seems higher for secreted proteins which do not only affect a single cell (Sun and Zhang 2020).

## Determinants and regulators of burst-like transcription

The mechanisms that underlie gene expression and burst-like transcription shall be discussed here in more detail. Permanent inactivation of specific genes or alleles is exerted by DNA methylation at CpG islands (CG-rich regions in promoters and in coding regions of genes). This epigenetic mechanism controls e.g. tissue specific inactivation of specific genes. DNA methyltransferases add methyl groups to cytosines within CpG islands. These methyl groups interfere with transcription factor binding to the DNA and thereby inhibit transcription. In addition, methylcytosine binding proteins (MBP) recruit histone-deacetylases and other co-repressors, finally increasing the chromosomal density and thereby forming transcriptionally inactive chromosomal structures (reviewed in (Attwood et al. 2002)). These structures do not take part in transcriptional bursting.

Adjustable regulation of gene expression shapes the bursts of transcription and occurs via alterations in the occupancy of chromosomes with nucleosomes, also called nucleosomal density. Nucleosomes are defined as chromosomal DNA which is wrapped around a histone octamer (Kornberg 1974). Genes with a high nucleosomal density are associated with numerous histones especially at the promoter sites and show lower transcription rates. In contrast, promoters of active genes show a lower nucleosomal density (reviewed in (Nicolas et al. 2017)). Key regulators of nucleosome density are histone modifications. Specific residues of histones can be methylated, acetylated, phosphorylated, and/or ubiquitinated (for a detailed review about modifications see (Bannister and Kouzarides 2011)). In brief, most kinds of acetylation, ubiquitination, and phosphorylation lead to reduced nucleosomal density and thereby to a higher transcriptional activity (Bannister and Kouzarides 2011). Histone methylation mainly increases the nucleosomal density, reducing transcription activity (Bannister and Kouzarides 2011).

Genes or alleles with a low nucleosomal density are accessible for transcription factors (TF) and RNA polymerase II (RNA-Pol II) that enable transcription (Li et al. 2007). Binding and release of TFs and RNA-Pol II occurs stochastically (Larsson et al. 2019; Urban and Johnston Jr. 2018), based on the thermodynamics of DNA and TF/RNA-Pol II interaction in the nuclear surrounding (Blake et al. 2006). The probability of TF and RNA-Pol II-binding increases with a decreasing nucleosomal density. In line with this, activated genes with a lower nucleosomal density show a higher burst frequency and burst size (duration) (Brown et al. 2013; Hornung et al. 2012b).

Based on this background, generation of transcriptional bursts can be described in a simplified way: to activate a gene, the nucleosomal density on the promoter is reduced. Random binding events of TFs and RNA-Pol II to the DNA lead to bursts of transcription; stochastic unbinding terminates the burst; rebinding will start another burst.

Burst-like transcription can be governed at different levels. We can distinguish between control of basal burst rates for specific genes and flexible modification of burst size and frequency to regulate gene expression. Basal burst rates seem to be determined mainly by regulatory sequences. Specific gene elements such as TATA boxes in the promoter recruit TFs and RNA polymerases. Genes, which encode for TATA boxes, show higher burst frequencies (Blake et al. 2006; Hornung et al. 2012a) and burst frequency and size depend on specific TATA box sequences (Hornung et al 2012a). Additionally, RNA-Pol II was shown to pause during transcription of a particular mRNA molecule and to continue after a certain time. This pausing and restarting was shown to underlie transcriptional bursts (Dobrzynski and Bruggeman 2009). It seems to be stochastic and can presumably be influenced by specific DNA sequences to affect burst size and frequency (Fujita et al. 2016). Flexible modifiers of bursts adjust gene expression to specific circumstances. Upregulated genes show e.g. decreasing nucleosomal density which will lead to a higher accessibility for TF and RNA polymerase and thereby a higher burst frequency and size (Kalo et al. 2015). The nucleosome and deacetylation remodeling complex (NuDR) was shown to fine tune gene expression by controlling the accessibility of TF and RNA-Pol II to the DNA (Bornelov et al. 2018). In addition, the availability of TFs and RNA-Pol II will shape the bursts. Higher levels of TFs may cause a higher burst frequency, as shown for cFos (Senecal et al. 2014). Moreover, binding probabilities of TFs on the DNA and thereby the bursts can be affected by trans-acting factors that modify the affinity of the TF for a particular promoter (Boettiger 2013).

## Assays to determine burst-like transcription

Different methods can be used to determine burst-like transcription. In fixed cells or tissues, single-molecule RNA fluorescence in situ hybridization (smRNA-FISH) can visualize actively transcribed alleles (Levesque et al. 2013). Probe sets that can bind to the pre-mRNA at the chromosomal locus and to the nascent mRNA are hybridized in fixed cells or tissue. Co-localization of the probe sets in the nucleus indicates an active transcription site (aTS), an actively transcribed allele (Levesque et al. 2013). Burst-like transcription, which is independent for the two alleles, is indicated by the occurrence of cells without, cells with one, and cells with two aTS in the same population. This method was used to determine active transcription sites in cultured cells (Levesque et al. 2013) and tissue sections from mouse kidney (Symmons et al. 2019). Our group provided evidence for burst-like transcription of the *MYH7* gene in cardiac tissue from HCM patients and non-transplanted donor hearts (Montag et al. 2018). Preliminary data indicate that *MYBPC3* and *TNNI3* are also expressed burst-like (Montag et al. 2019).

In living cells, fluorescently tagged mRNAs can be used to examine kinetics of burst-like transcription over time. Here, specific stem loop sequences are inserted by genome editing to the 3′- or 5′-end of the mRNA of interest. Fluorescently labeled bacterial proteins that can bind to the specific stem loop sequences are co-expressed in these cells and fluorescent signals indicate whether the respective RNA is transcribed. Live cell imaging then directly visualizes transcription of the mRNA molecules in the nuclei and the stochastic on and off switch of the gene of interest (Darzacq et al. 2007; Yunger et al. 2010).

**Fig. 2:**
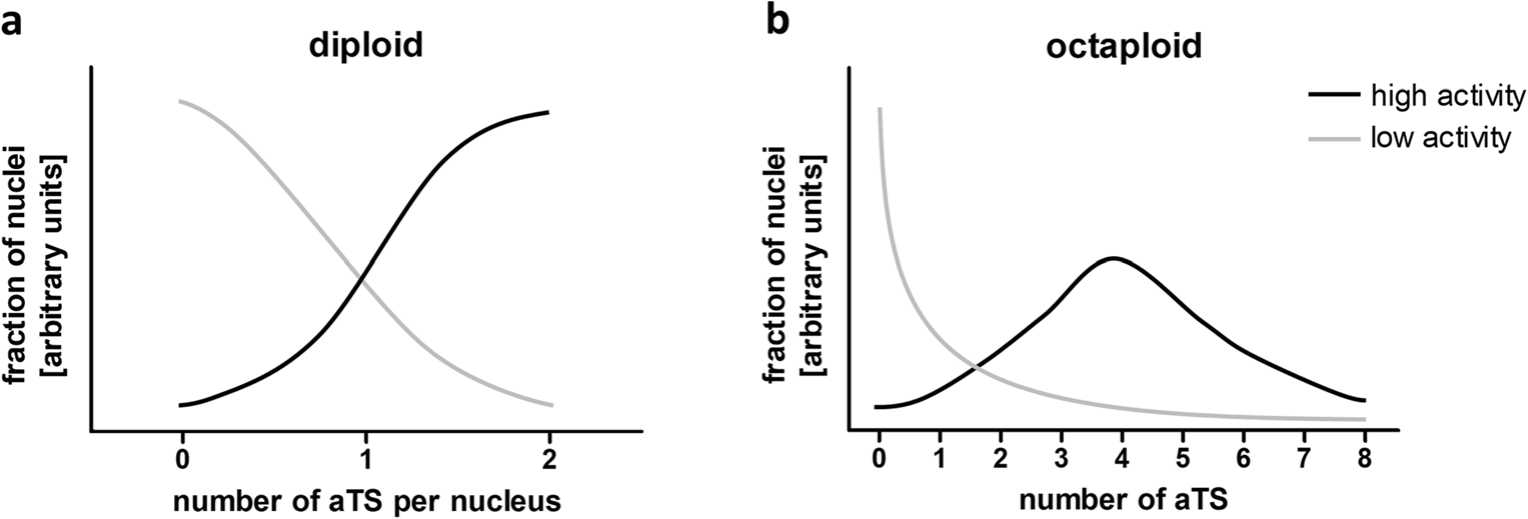
Burst-like transcription of alleles leads to stochastic distribution of active transcription sites (aTS) per nucleus. During phases with high activity, the probability for each allele to be active increases; thus, more nuclei will show more aTS. The graphs depict estimated fractions of aTS per nuclei in highly and lowly expressed genes. **a** In diploid cells, genes with a low activity will provide mostly nuclei without aTS and in genes with high activity most nuclei will show two aTS. **b** In polyploidy cells, low activity genes will again show mostly nuclei without aTS. Highly active genes in polyploid cells will also show a higher number of nuclei with more than one aTS. According to our results and published data on the ploidy of cardiomyocytes (Herget et al. 1997; Montag et al. 2018), we assume that most cells are tetraploid; therefore, the maximum of nuclei will show four aTS

The activity of a respective gene is most likely correlated with the number of aTS per nucleus. In highly active genes, the bursts will occur more frequently and have a longer duration (Dar et al. 2012). This will result in a higher percentage of cells that contain one or two aTS. In contrast, genes with a low activity will show high percentage of cells without aTS and more cells with only one aTS (Fig. 2a). This will also apply to polyploid cells that contain more than 2n of chromosome sets. In two independent studies where the ploidy of healthy adult human cardiomyocytes was determined, 19 or 22% of the nuclei were diploid, 45 or 60% tetraploid, 2% hexaploid, 15% or 23% octoploid, 1 or 11% 16-ploid, and 0 or 1% were 32-ploid (Herget et al. 1997; Montag et al. 2018). Increases in ploidy are found in cardiac diseases such as myocardial infarction and HCM (Herget et al. 1997; Montag et al. 2018; Shliakhto et al. 2007; Sukhacheva et al. 2015). In line with this, we have determined nuclei with more than two *MYH7*-aTS for both donor and HCM patients (Montag et al. 2018). Genes with a low activity will show mostly nuclei without aTS and few nuclei with more than two aTS. In contrast, most nuclei from highly active genes will show more than two aTS. For cardiomyocytes, where most cells are tetraploid, we expect the maximum at 4 aTS per nucleus (Fig. 2b).

## Burst-like transcription leads to allelic imbalance from cell to cell

**Fig. 3:**
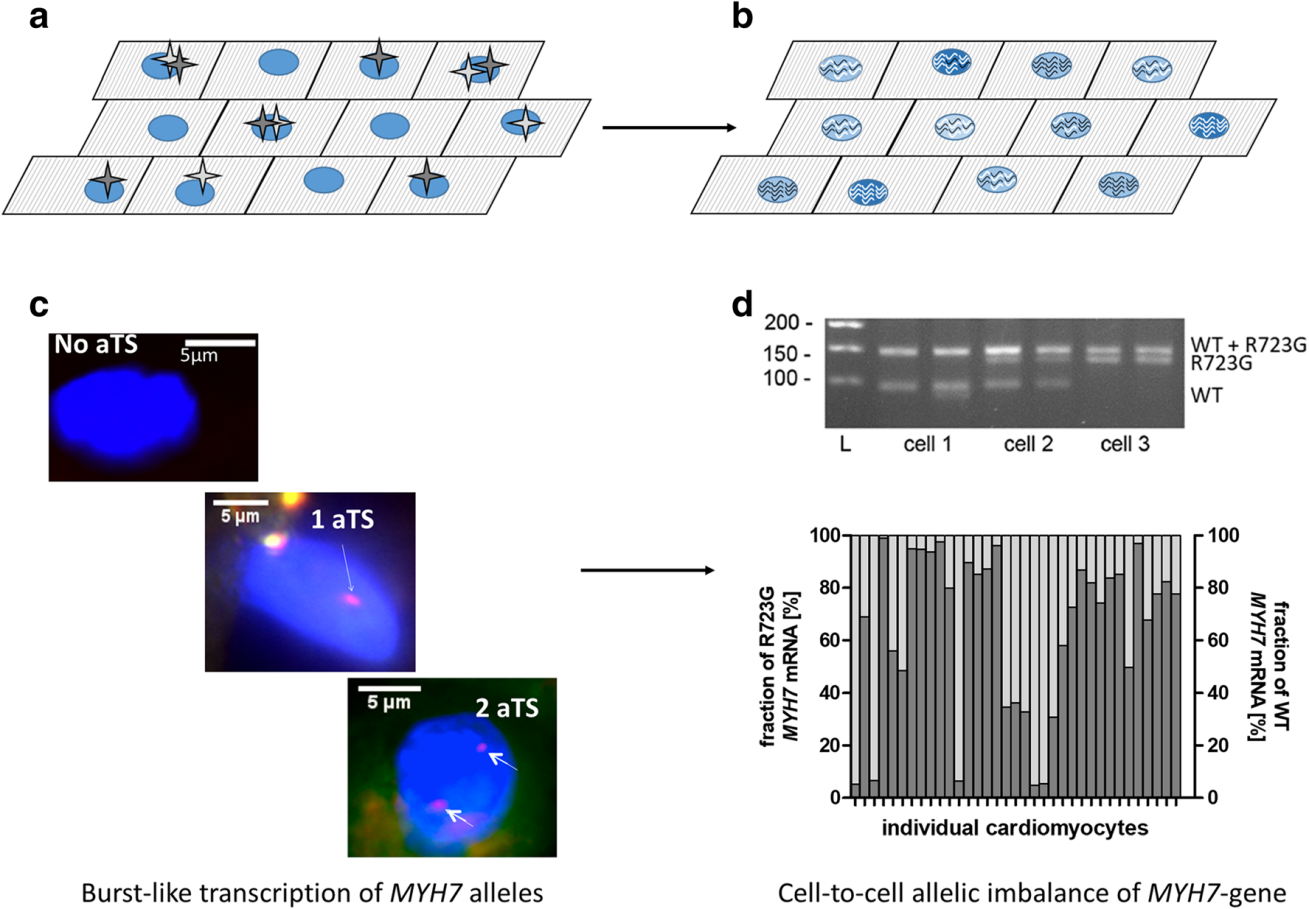
Burst-like transcription of *MYH7* leads to allelic imbalance from cell to cell. **a** Schematic of cardiomyocytes within cardiac tissue with active transcription sites (aTS) for allele A (wild-type; light gray star) and allele B (mutant; dark gray star). The tissue contains cells that at the given time point transcribe both alleles, no allele, only allele A, and only allele B. **b** Accumulation of mRNA molecules from bursting of the two alleles leads to different ratios of wild-type vs. mutant mRNA from cell to cell, ranging from cells with mainly wild-type allele A (light gray waves), mainly mutant allele B (dark gray waves), and mixtures of both alleles. **c** Using smRNA-FISH, we visualized active transcription sites (aTS) in nuclei from an HCM patient with the *MYH7* mutation R723G. We found nuclei without aTS (top), cells with one aTS (middle), and cells with more than one aTS (here, two aTS, bottom). This indicates burst-like, independent transcription of the two *MYH7* alleles. **d** In individual cardiomyocytes isolated by laser-microdissection from cryosections of myocardial samples from the same patient, we detected highly variable fractions of mutant *MYH7* mRNA from cell to cell as revealed by single-cell RT-PCR and allele-specific restriction analysis (top). Densitometric quantification of mutant and wild-type mRNA fractions showed substantial allelic imbalance from cell to cell, with cells that express essentially only mutant mRNA, cells that express almost only wild-type mRNA, and cells with mRNA from both alleles in different ratios (bottom). Figures from **c** and **d** are reprinted and modified from (Montag et al. 2018), with permission from Frontiers

So far, we have discussed burst-like transcription at the gene level. Importantly, the described mechanisms that underlie burst-like transcription also lead to a stochastic expression of the alleles over time, meaning each allele is switched on and off independently from the other one. Therefore, in cells from the same population, at a given time point, either allele A alone can be active, allele B alone can be active, both alleles can be active, or no allele can be active (Fig. 3a). This induces an allelic imbalance from cell to cell (Fig. 3b); in the same population, some cells contain high fractions of mRNA from allele A, some contain high fractions of mRNA from allele B, and others contain mRNA from both alleles in different ratios (Montag et al. 2018; Raser and O’Shea 2005; Symmons et al. 2019).

Initial studies termed this phenomenon *random monoallelic expression*. They could show that the expression of the alleles can differ between the cells from the same population (Reinius and Sandberg 2015). However, in contrast to the association that may be evoked by the term “monoallelic,” the authors also detected cells, which expressed both the maternal and the paternal allele. This detection of mono- and biallelic mRNA expression led to the assumption of a dynamic on and off switch, a stochastic choice of the two alleles (Deng et al. 2014; Levesque et al. 2013; Reinius and Sandberg 2015). With the emergence of single-cell RNA sequencing techniques, the unequal allelic expression from cell to cell is determined in more and more different cell types (Borel et al. 2015; Deng et al. 2014; Sun and Zhang 2020).

Importantly, stochastic on- and off-switching of the alleles over time may lead to accumulation of mRNA from previous bursts depending on the particular mRNA turnover rates. Thus, cells without aTS may contain mRNA molecules of this gene and cells with only allele A being active still may contain mRNA molecules from allele B. In addition, the allelic expression within one cell will change over time. Therefore, a cell with mainly mRNA from allele B can express high levels of mRNA from allele A at another time point. The mechanisms described for regulation of bursts will also affect unequal allelic mRNA distribution from cell to cell. Higher burst rates will lead to a higher activation rate of both alleles and may thus reduce the allele-specific variability between individual cells from one population (Kalo et al. 2015).

For the *MYH7* gene, we have recently provided evidence that burst-like transcription of mutant and wild-type alleles could lead to allelic imbalance from cell to cell. In cardiomyocytes from HCM patients with different *MYH7* mutations, we observed burst-like transcription of the two *MYH7* alleles by smRNA-FISH (Fig. 3c) and highly variable fractions of mutant and wild-type mRNA from cell to cell (Fig. 3d) (Montag et al. 2018). Model calculations were used to test our hypothesis that burst-like transcription underlies the observed allelic imbalance from cell to cell (Montag et al. 2018). Indeed, using the fraction of cardiomyocytes with active transcription sites from our experiments and published *MYH7*-mRNA turnover rates as an input for the model, the calculated variability of allelic expression of mutant and wild-type β-MyHC among individual cardiomyocytes was comparable to our experimental data. Thus, we assume that the *MYH7* transcriptional bursts underlie the observed unequal allelic expression from cell to cell (Montag et al. 2018). In line with our research, a recent study shows burst-like transcription of maternal and paternal alleles for different genes in mouse kidney and relates this to cell-to-cell allelic imbalance of these genes (Symmons et al. 2019).

## Translation of unequal allelic mRNA fractions from cell to cell into unequal fractions of mutated and wild-type protein

To date, quantification of mutant and wild-type β-MyHC at the single-cell level is not possible due to technical limitations. Model calculations showed comparable variable fractions of mutant and wild-type alleles from cell to cell at mRNA and protein level (Montag et al. 2018). The assumption that variable fractions of mutated mRNA from cell to cell correspond to a similar variability also at the protein level is strongly supported by the large functional variability among individual HCM cardiomyocytes (Kraft et al. 2016; Montag et al. 2018). Additional evidence for variable allele-specific protein distribution from cell to cell is provided by independent studies that showed a patchy distribution of wild-type cMyBP-C protein from cell to cell and even within individual cardiomyocytes of HCM patients with cMyBP-C truncation mutations (Aldag-Niebling et al. 2018; Kraft and Montag 2019; Parbhudayal et al. 2018; Theis et al. 2009). The truncations lead to haploinsufficiency; thus, the remaining protein in the sarcomeres originates just from the wild-type allele. Only periods with bursts from the wild-type allele will therefore lead to (overall reduced) expression of functional protein, which may underlie the patchy distribution of cMyBP-C from cell to cell. In addition, a recent publication shows that *MYBPC3*-mRNA is transported to the Z-disc where it is translated and incorporated into the sarcomeres (Lewis et al. 2018). If the mRNA from a burst of one allele is transported to a certain area of a cell, this may lead to the observed uneven distribution of wild-type cMyBP-C within individual cardiomyocytes (Aldag-Niebling et al. 2018; Kraft and Montag 2019; Theis et al. 2009). Taken together, model calculations as well as uneven distribution of wild-type cMyBP-C and functional variability from cell to cell strongly suggest that allelic mRNA-imbalance translates into imbalance of wild-type and mutated protein from cell to cell.

## Consequences of allelic imbalance from cell to cell: the contractile imbalance hypothesis and HCM pathogenesis

In heterozygous individuals where mutant and wild-type alleles encode for proteins with different functional properties, it will affect the function of the cell from which allele the protein is translated at a specific time point. For mutations in secreted proteins, the impact on each individual cell may be small since all cells in the vicinity will be exposed to a comparable mixture of wild-type and mutated proteins (Sun and Zhang 2020). In HCM patients with mutations that alter force generation as shown for *MYH7* mutations (Bloemink et al. 2014; Kirschner et al. 2005; Seebohm et al. 2009; Sommese et al. 2013), different fractions of mutant and wild-type sarcomeric proteins from cell to cell may lead to different phenotypes of individual cells. Such a phenotypic heterogeneity could increase disease severity in addition to primary functional effects of the mutation itself (Deng et al. 2014).

**Fig. 4:**
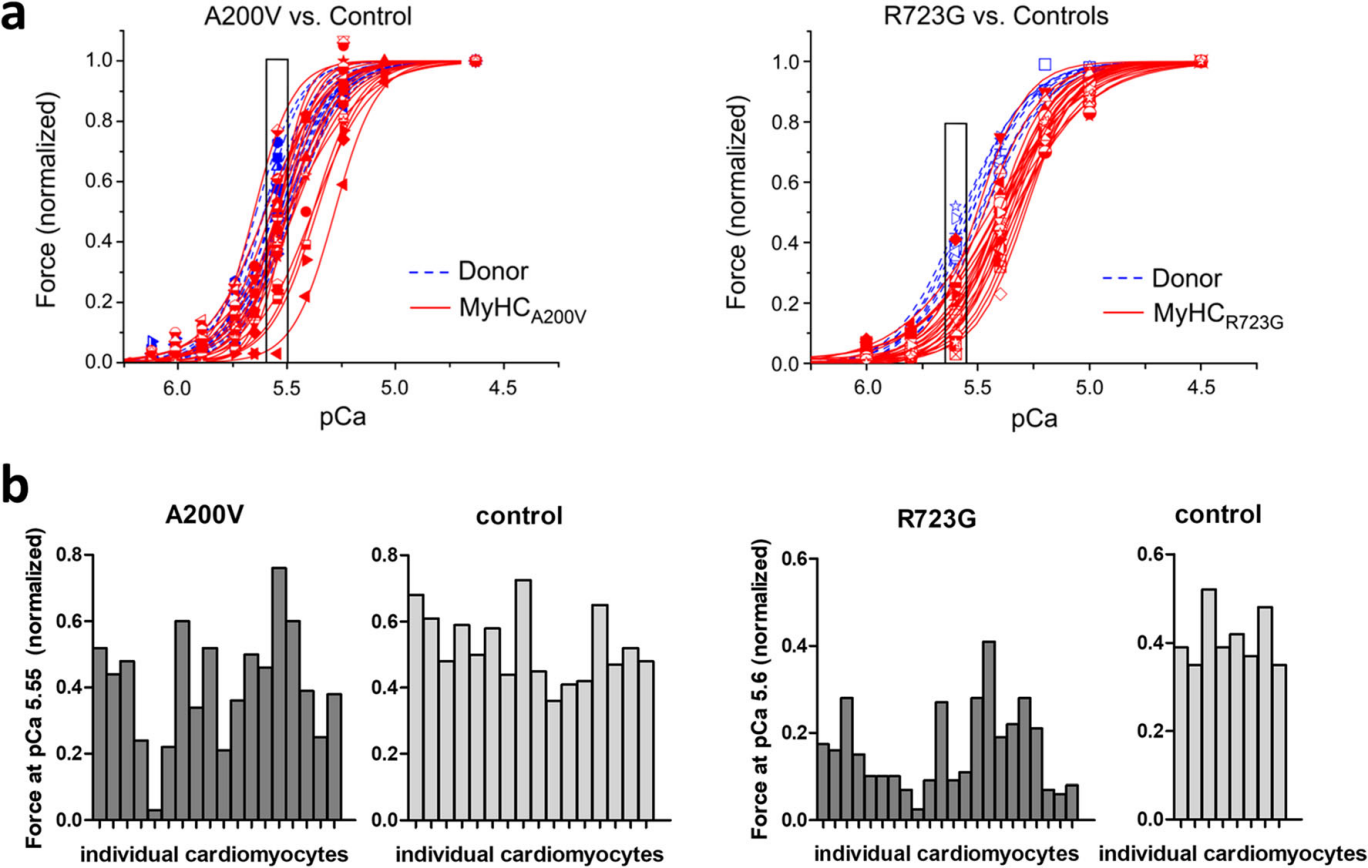
Contractile imbalance in HCM patients with mutations in β-MyHC. Single permeabilized cardiomyocytes, isolated from heart tissue of HCM patients (red) with the mutation A200V (left) or R723G (right), respectively, and from donor myocardium (blue) as controls were adjusted for phosphorylation levels. Cardiomyocytes were subjected to different calcium concentrations to measure isometric force generation. **a** Forces of each individual cardiomyocyte were normalized to maximum force and plotted against the respective calcium concentration (force-pCa-relations). Each symbol and curve represents a different individual cell. **b** Relative forces at physiological calcium concentrations during twitch (indicated by boxes in **a**) were plotted for individual cardiomyocytes from patients and controls. Whereas for controls, force generation varied no more than twofold, the patient’s cardiomyocytes showed a much larger variability of 10 to 20-fold. Data were published in Montag et al. (2018); **a** is reprinted and modified from (Montag et al. 2018), with permission from Frontiers

To characterize functional effects of HCM mutations in β-MyHC, we analyzed calcium-dependent force generation in isolated, permeabilized single cardiomyocytes from HCM patients with mutations A200V and R723G compared with donor controls (Kraft et al. 2016; Montag et al. 2018). Mutation A200V is located in the myosin head domain, close to the nucleotide-binding pocket. Mutations in this region are assumed to affect the ATPase activity of β-MyHC (Colegrave and Peckham 2014). Mutation R723G is located in the converter region of β-MyHC. The mutation leads to an increased stiffness of the converter and thereby increased force generation (Seebohm et al. 2009). Taking together all analyzed cells, respectively, both mutations lead to a right shift of the force-pCa-curve, indicating calcium desensitization. However, when looking at the individual force-pCa curves of the HCM cardiomyocytes for each mutation, we found a rather large variability in calcium sensitivity and thus in force generation at physiological calcium concentrations (Fig. 4). This variability was much larger in HCM cardiomyocytes than in donor cells. Whereas some HCM cardiomyocytes showed relative force generation comparable to donor cells, other cardiomyocytes from the very same patient showed a 10–20-fold reduced force at the identical calcium concentration (Fig. 4). We named this functional variability among individual cardiomyocytes *contractile imbalance*. Preliminary data suggest that a missense mutation in cTnI and a truncation mutation in cMyBP-C also lead to contractile imbalance (Montag et al. 2019).

## Implications and limits of the contractile imbalance hypothesis

The finding that individual cardiomyocytes generate different forces at the same calcium concentration may have a severe impact on the physiological function of the myocardium. Our results suggest that the coordinated contraction of neighboring cardiomyocytes within the functional cardiac syncytium may be disturbed in the heart of HCM patients. During each twitch, contracting neighboring cardiomyocytes will generate different forces. This will render them unable to act uniformly. Stronger cells in the myocardium may over-contract, whereas weaker cells may be over-stretched, finally disrupting the myocardial network. Over the years while the phenotype develops, contractile imbalance may thereby exacerbate or lead to the typical, HCM-associated myocardial disarray.

As derived from model calculations, burst-like transcription which is independent for the two alleles will lead to varying fractions of mutated and wild-type mRNA and protein within each cell over time. Thus, cardiomyocytes with high fractions of wild-type protein and therefore almost normal function can convert into cells with high fractions of mutant protein (or low levels of functional protein in case of truncation mutations) and highly altered function at another time point. Repeatedly altering function of individual cardiomyocytes may further contribute to disruption of the syncytium and thereby to development of cardiomyocyte and myofibrillar disarray.

Increased stretch in cultured cardiomyocytes induces release of TGF-β, angiotensin II, and endothelin-1 (Ruwhof et al. 2000; van Wamel et al. 2001) and expression of hypertrophy marker genes (van Wamel et al. 2002). In a HCM mouse model, increased strain due to the mutation was associated with increased TGF-β expression and activated pro-fibrotic pathways and hypertrophic remodeling (Teekakirikul et al. 2010). In other mouse models, expression of TGF-β activated fibroblasts and myofibroblasts and thereby induced cardiac fibrosis and hypertrophy (Khalil et al. 2017). Accordingly, we assume that contractile imbalance in HCM patients may lead to myocardial disarray and release of TGF-β, finally triggering hypertrophy and fibrosis (Brenner et al. 2014).

Notably, substantial alteration of sarcomere function and the force generating mechanism by HCM mutations is pathologic for the myocardium per se, as can be seen from very rare homozygous HCM patients where a mutation-induced contractile imbalance cannot be expected. Interestingly, these patients show a different and mostly more severe course of disease compared with their heterozygous relatives (Nishi et al. 1994). In homozygous HCM patients, the disease may be aggravated due to a gene dosage effect, as discussed in more detail in Brenner et al. (2014). Another hypothesis suggests a mechanism which may underlie a non-uniformity between cardiomyocytes in heterozygous and homozygous patients. It assumes that the mutation-induced disturbed protein function may affect the development of the contractile system in early stages of life and induce different degrees of myofibrillar disarray from cell to cell early on (Mansson 2014).

## Conclusions

We have shown that the alleles of the *MYH7* gene are transcribed in bursts, most likely causing allelic imbalance from cell to cell. The unequal fractions of mutated and wild-type β-MyHC are associated with contractile imbalance among the individual cardiomyocytes; at the same calcium concentration, cardiomyocytes generate highly different forces. This may disrupt the myocardial syncytium and trigger cardiomyocyte disarray, a hallmark of HCM. In addition, the variable forces may induce pro-hypertrophic and pro-fibrotic pathways in HCM patients. We assume that this mechanism may apply for all HCM mutations that alter force generation, irrespective of the kind of alteration or the underlying mechanism.

## Data Availability

Not applicable.
